# Pelvic Hematoma With Acute Hemorrhage Secondary to Inferior Vena Cava Filters

**DOI:** 10.7759/cureus.46652

**Published:** 2023-10-07

**Authors:** Pallavi Pokharel, Fawaz Araim, Faith Adekunle

**Affiliations:** 1 Department of Surgery, St. Agnes Hospital, Baltimore, USA; 2 Medicine, School of Medicine, American University of the Carribbean, Cupecoy, SXM

**Keywords:** inferior vena cava, inferior vena cava filter (ivcf), retroperitoneal hematoma, ivc thrombectomy, pelvic hematoma, deep vein thrombosis (dvt)

## Abstract

Recurrent deep vein thrombosis (DVT) and inferior vena cava (IVC) thrombosis are well-known complications of inferior vena cava filters (IVCFs); however, pelvic hematoma is a rare finding. In this study, we present a case of a 41-year-old female who presented with severe abdominal pain. Extensive bilateral lower extremity DVT, thrombosis extending up to the level of IVCF, and a pelvic hematoma with acute hemorrhage were diagnosed. Mechanical thrombectomy of the IVC, bilateral iliac, and femoral veins with stent placement in the external iliac veins was performed under general anesthesia. This rarely reported case remains a challenge to diagnose and treat because of its complex mechanisms and multiple risk factors. Our case highlights the importance of the surgical strategy adopted and the need for a good initial assessment.

## Introduction

Venous thromboembolism (VTE), deep vein thrombosis (DVT), and pulmonary embolism (PE) affect one to two individuals per 1,000 annually [1]. Even though anticoagulation is the preferred treatment, inferior vena cava filter (IVCF) is another therapeutic option in the management of VTE when anticoagulation is contraindicated. IVCFs can cause significant morbidity and, in rare instances, mortality. A few delayed complications seen are filter migration to the heart and other locations, bleeding, inferior vena cava (IVC) perforation, and filter fractures [2]. With the unpredictability of the bleeding to rapidly exsanguinate and cause shock, the patient’s survival is highly dependent on early diagnosis. High clinical standards of care and early screening methods are essential to prevent these complications and the morbidity and mortality associated with each. We report the case of an IVCF-related delayed lower extremity DVT and retroperitoneal hematoma in a patient with a medical history of motor vehicle accident requiring IVCF placement due to acute contraindication to anticoagulants.

## Case presentation

**Figure 1:**
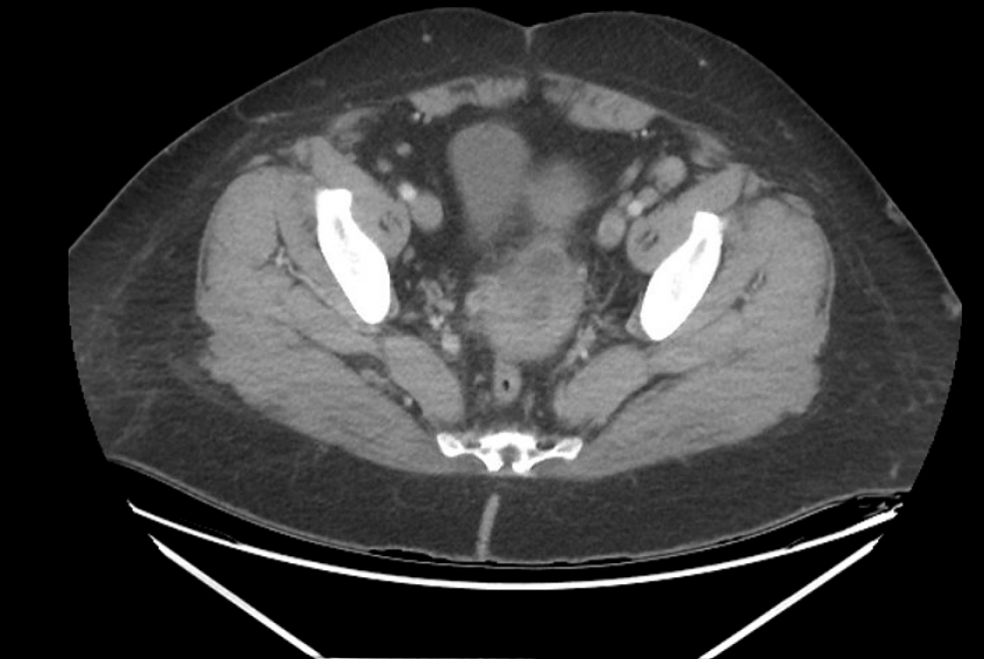
Axial CT image of the abdomen/pelvis with IV contrast demonstrating no hematoma. CT, computed tomography

A 41-year-old Caucasian female with a history of prior DVT, motor vehicle collision 20 years ago with emergent sacral screw placement, cesarean section, splenectomy, and unclear history of IVCF placement presented to the emergency department (ED) with worsening abdominal and back pain and vaginal spotting. To note, the patient presented to the ED a day prior with similar symptoms with normal computed tomography (CT) (Figure 1) findings and was sent home on oral antibiotics.

**Figure 2:**
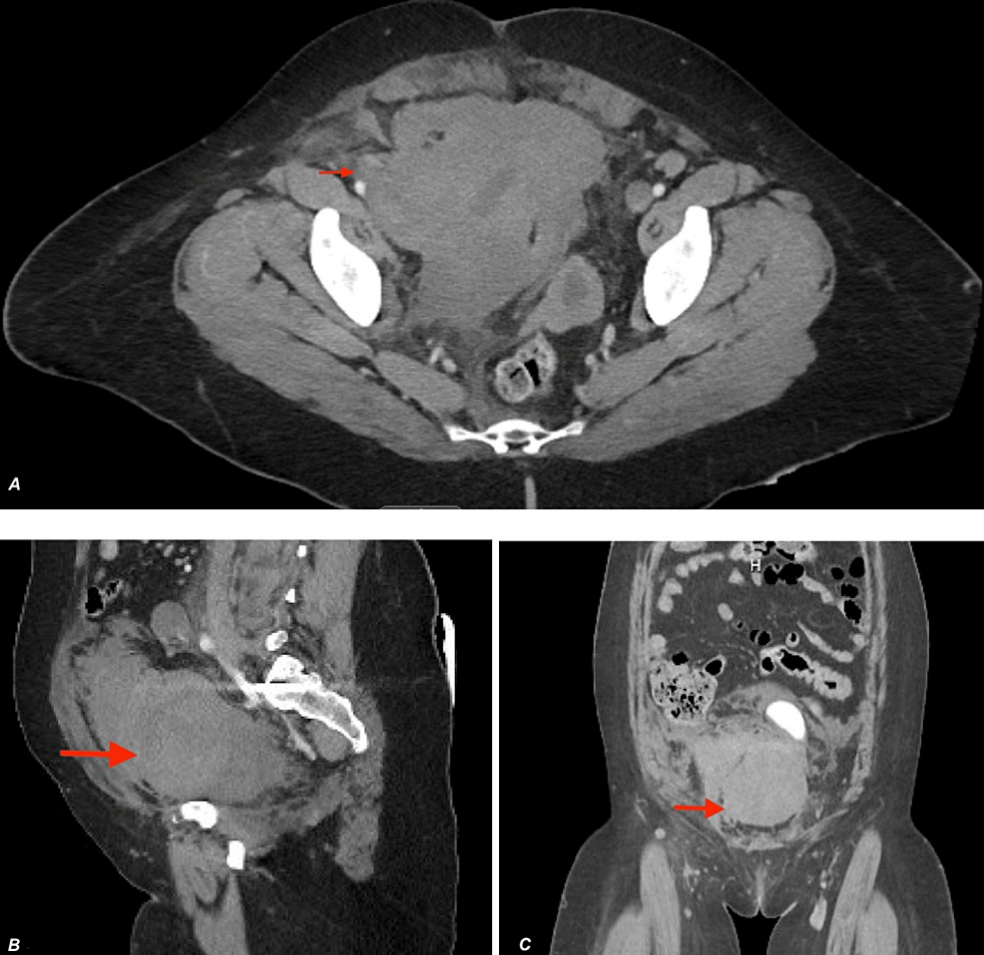
Axial (A), sagittal (B), and coronal (C) CT angiography images of the lower extremity demonstrating hematoma. CT, computed tomography

**Figure 3:**
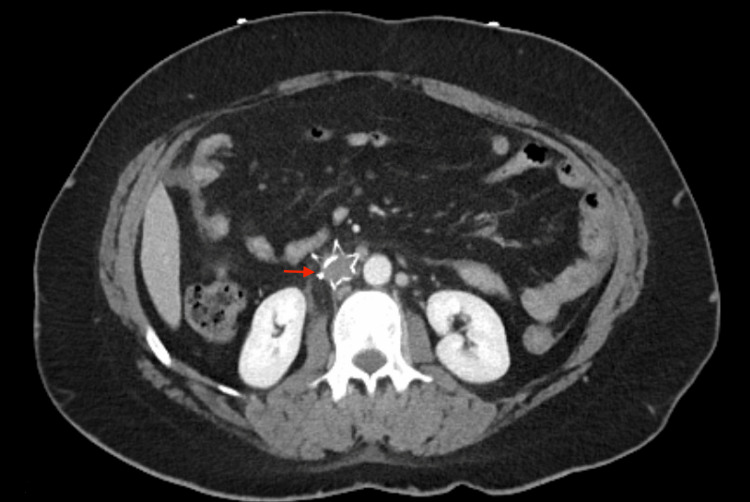
Axial CT angiography image of the lower extremity demonstrating the IVC filter. CT, computed tomography; IVC, inferior vena cava

**Figure 4:**
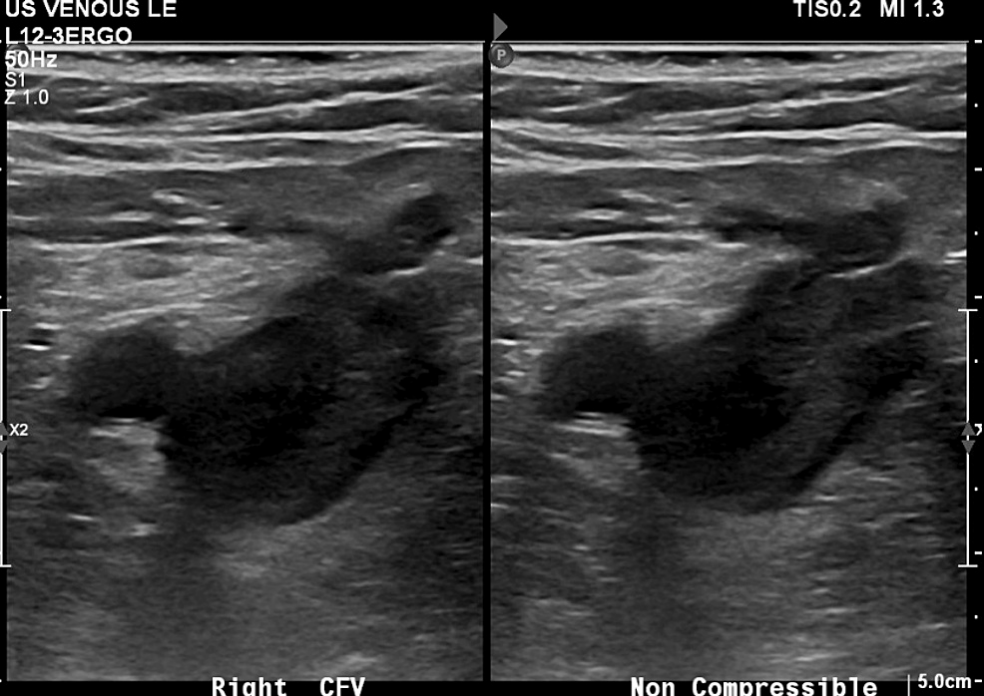
Lower extremity venous duplex demonstrating DVT of the right common femoral vein. DVT, deep vein thrombosis

Upon arrival to the ED, the patient’s vital signs were within the normal limits. Physical examination by an ED physician was notable for a non-distended, diffusely tender lower abdomen on palpation. The patient exhibited pain when bearing weight on the bilateral limb with no evidence of phlegmasia, numbness, or weakness. Bilateral lower extremities were distally warm and well-perfused. There was a mild right lower extremity swelling with tenderness in the calf. Initial laboratory studies revealed a white blood cell count of 23.1 K/uL, hemoglobin of 8.3 g/dL, and lactic acid of 3.3. The remainder of the laboratory basic metabolic panel results did not reveal any abnormalities. The electrocardiographic and chest radiographic findings were unremarkable. Computed tomography angiography (CTA) of the lower extremity showed a likely interval development of hematoma measuring 12.4 x 12.4 cm (Figure 2) in size in the pelvis, as well as acute hemorrhage without evidence of extravasation. Thrombosis of the right popliteal vein extending superiorly into the IVC at the level of the IVCF can also be seen (Figure 3). Venous duplex ultrasound of the bilateral lower extremities revealed diffuse, extensive VTE in the bilateral lower extremities involving the external iliac, common femoral, profunda femoris, femoral, popliteal, and posterior tibial veins (Figure 4). Surgical consultation was sought at this time. 

Findings of the physical examination were significant for lower abdominal tenderness and bilateral lower extremity 3+ pitting edema. On the Doppler scan, she had biphasic signals to the right dorsalis pedis and bilateral posterior tibial vein. Additionally, she had triphasic signals to the left dorsalis pedis.

The patient was briefly started on heparin while in ED, but medication was put on hold due to the finding of hematoma on CTA. The patient was admitted for observation. The patient was further managed with elevation, compression, serial labs, and bed rest. During her hospital stay, given her downtrending hemoglobin level of 6.7 g/dL, multiple units of packed red blood cells were given. With no changes on repeated CTA of the abdomen/pelvis and stable hemoglobin, the patient was resumed on heparin drip on hospitalization day (HD) 4.

**Figure 5:**
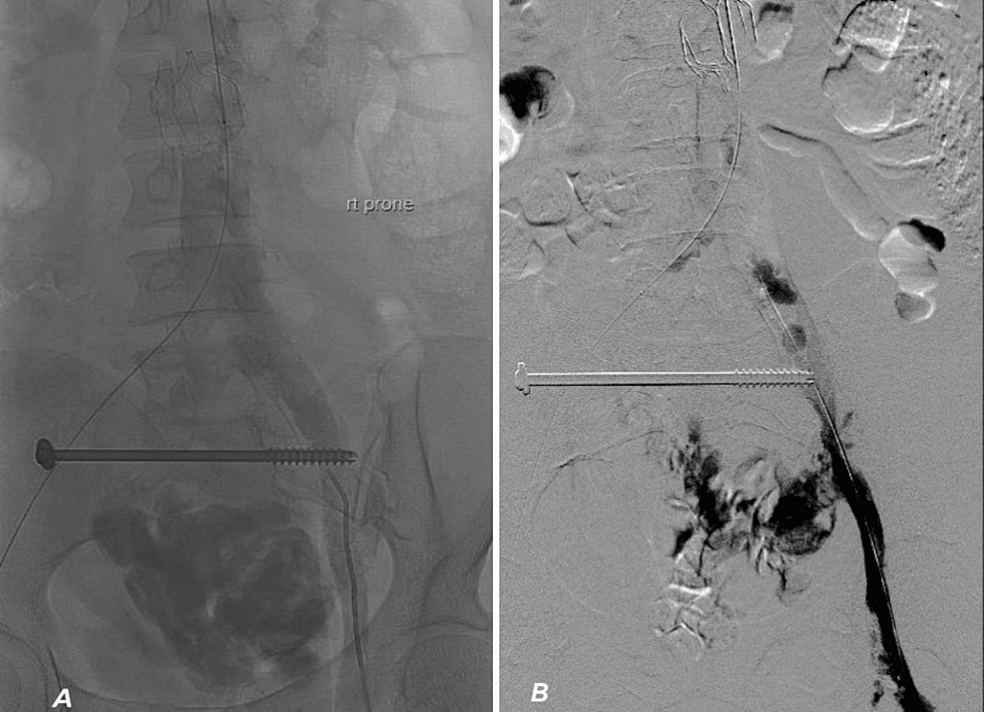
Abdominal arteriogram demonstrating hematoma of the external iliac vein.

**Figure 6:**
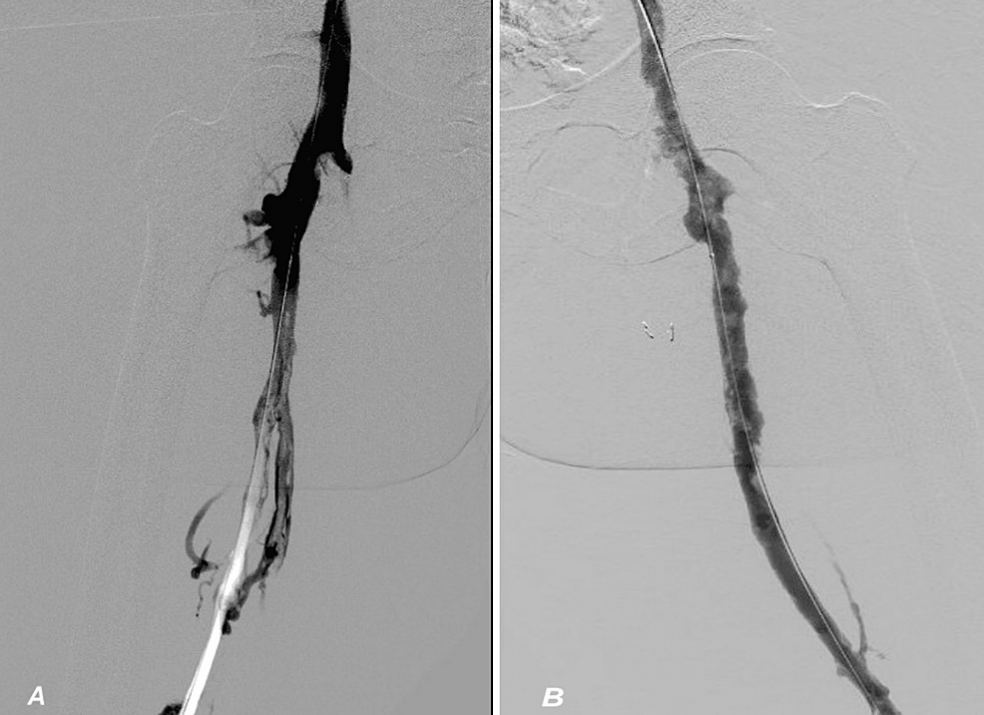
Abdominal arteriogram demonstrating extensive deep vein thrombosis in the bilateral common femoral vein and the femoral vein.

The patient’s hemoglobin stayed stable after resuming the heparin drip. On HD 6, venogram of the bilateral popliteal, femoral, and iliac veins, intravascular ultrasound, and thrombectomy of the bilateral femoral, external iliac, common iliac, and inferior vena cava veins using penumbra device (lightning bolt) were performed, followed by angioplasty of the right external iliac vein with stent (LifeStream, Becton Dickinson, Franklin Lakes, NJ) placement and angioplasty of the left common femoral and external iliac veins (Figures 5, 6).

Her postoperative course was uneventful. Postoperatively, the patient's pain was controlled, and the patient tolerated a diet. The patient was transitioned to oral anticoagulation. Throughout her hospital stay, labs revealed leukocytosis, likely reactive, as other causes of infection were ruled out. The patient was discharged on HD 8 in stable condition with a rolling walker.

## Discussion

IVCFs are commonly placed in patients with a high risk of DVT or blood clots who are unable to tolerate blood thinners. Complications of IVCFs include infection, PE, occlusion of IVC, and filter migration [3].

Retroperitoneal hematoma is a rare complication of IVCFs. It is seen to have an incidence of 0.1% to 0.6% in patients receiving oral anticoagulants [4]. This is a fatal complication mostly when undergoing anticoagulant and thrombolytic therapy. Some patients are asymptomatic, while others tend to present with groin, abdominal, and back pain, or hemodynamic instability [5]. Due to the rare occurrence, the pathophysiology related to the spontaneous bleeding is unclear, but a hypothesis has been made that heparin or anticoagulation-induced immune microangiopathy could be involved [4].

It is imperative that patients presenting with signs and symptoms of a retroperitoneal hematoma be evaluated as soon as possible due to the potential for death associated with this complication. It is important to note that the patient's hemodynamic stability will determine the course of treatment and the workup. Hemodynamically stable or asymptomatic patients should undergo a CT/CTA, whereas hemodynamically unstable patients should undergo an angiography. A decision should be made regarding the mode of intervention once the point of bleeding has been determined and whether it is an arterial or venous bleed. In the case of large arterial and venous bleeds, immediate surgical intervention would be necessary, but the presence of slow venous bleeds from small vessels may be observed with continuous laboratory monitoring [4]. This is determined by how stable the patient is and what is seen on imaging.

## Conclusions

DVTs, IVC thrombosis, and retroperitoneal hematoma are complications of IVCFs. Events can present acutely or chronically after the IVCF placement. Depending on hemodynamic stability, the patient should promptly undergo surgical intervention or appropriate imaging CT/CTA. Once stable, anticoagulation should be started while closely monitoring the hematoma for further evidence of extravasation. For similar clinical scenarios, treatments should be tailored to each patient depending on their overall clinical situation.
